# Egg Intake and the Incidence of Alzheimer’s Disease in the Adventist Health Study-2 Cohort Linked with Medicare Data

**DOI:** 10.1016/j.tjnut.2026.101541

**Published:** 2026-04-17

**Authors:** Jisoo Oh, Keiji Oda, Gabriela Chiriac, Gary E Fraser, Rawiwan Sirirat, Joan Sabaté

**Affiliations:** 1 Center for Nutrition, Healthy Lifestyles, and Disease Prevention, School of Public Health, Loma Linda University, Loma Linda, CA, United States; 2 Adventist Health Study, Research Affairs, Loma Linda University, Loma Linda, CA, United States; 3 Department of Medicine, School of Medicine, Loma Linda University, Loma Linda, CA, United States

**Keywords:** Adventist Health Study-2, egg intake, Alzheimer’s disease, Medicare, cohort study

## Abstract

**Background:**

A substantial knowledge gap remains regarding the relationship between modifiable dietary factors and Alzheimer’s disease risk. Eggs are a source of key nutrients that support brain health.

**Objectives:**

The aim of this study was to investigate the association between egg consumption and the incidence of Alzheimer’s disease.

**Methods:**

Data were drawn from the Adventist Health Study-2, a large, prospective cohort of United States Seventh-day Adventists, linked with Medicare records to identify Alzheimer’s disease diagnosis. Diet and lifestyle factors were assessed using a validated food frequency questionnaire. Egg consumption was categorized by frequency, ranging from never/rarely to ≥5 times per week. The analytic sample included 39,498 participants (mean follow-up: 15.3 y), among whom 2858 developed Alzheimer’s disease. Multivariable-adjusted Cox proportional hazards models estimated hazard ratios (HRs) and 95% confidence intervals (CIs). Restricted cubic spline analysis was conducted using continuous egg intake (g/d).

**Results:**

Egg consumption was inversely associated with Alzheimer’s disease risk. Compared with never/rarely consuming eggs, HRs (95% CIs) after adjusting for demographic, lifestyle, food groups, and comorbidities were 0.83 (0.75, 0.92) for 1–3 times per month, 0.83 (0.74, 0.94) for once per week, 0.80 (0.71, 0.90) for 2–4 times per week, and 0.73 (0.60, 0.89) for ≥5 times per week. In the spline model, zero egg intake was curvilinearly associated with an adjusted HR of 1.22 (1.11, 1.34) compared with 10 g/d.

**Conclusions:**

In this health-conscious population, moderate egg consumption was associated with a significantly lower risk of Alzheimer's disease. These findings suggest a potential neuroprotective benefit of nutrients found in eggs when consumed as part of a balanced diet.

## Introduction

Alzheimer’s disease is a progressive neurodegenerative disorder characterized by the accumulation of amyloid-β plaques, neurofibrillary tangles, and widespread neuronal loss, ultimately leading to cognitive decline, loss of independence, and death [1]. In the United States, it is the sixth leading cause of death. The economic impact is substantial, with national costs for managing Alzheimer’s disease projected to exceed $600 billion annually by 2050 [2]. During the same period, the proportion of Americans aged ≥65 y is expected to double from ∼10% to 20%, amplifying the public health burden [3].

Although the etiology of Alzheimer’s disease is multifactorial, key contributors include genetic predisposition, vascular health, and environmental exposures, particularly diet. In the absence of curative treatments and given the limited efficacy of current pharmacological therapies, prevention strategies targeting modifiable risk factors have become increasingly important [4,5]. Our previous research found that vegetarians had higher cause-specific mortality from dementia compared with nonvegetarians, whereas a vegetarian diet was associated with reduced risk of all-cause and several cause-specific mortalities [6].

Emerging evidence suggests a potential role for egg consumption in modifying Alzheimer’s disease risk [7]. Moderate egg intake has been associated with a 10% reduction in mortality from neurodegenerative disease [8]. Although some studies have reported cognitive benefits associated with egg consumption, most have been limited by short follow-up periods, cross-sectional designs, or reliance on self-reported cognitive outcomes [[9], [10], [11]]. These limitations hinder the ability to establish causality or capture the progression to clinically diagnosed Alzheimer’s disease. In addition, existing studies vary widely in how they assess egg intake, and few incorporate biomarker validation or clinically confirmed endpoints. Inconsistent adjustment for comorbidities and socioeconomic factors also introduces potential confounding, highlighting the need for more rigorous investigation.

The Adventist Health Study-2 (AHS-2) cohort offers a unique opportunity to explore this relationship in a well-characterized population with a broad range of egg intake. Given its detailed dietary assessments and linkage with Medicare claims data, this cohort is particularly well-suited to examine the association between egg consumption and incident Alzheimer’s disease, independent of other dietary and lifestyle factors. Therefore, the study aimed to investigate the relationship between egg intake and Alzheimer’s disease risk in the AHS-2 cohort, adjusting for key confounding variables.

## Methods

### Study participants and design

This study used data from the AHS-2 cohort, linked with Medicare claims data files. AHS-2 is a large, long-term, population-based prospective cohort study that enrolled >96,000 members of the seventh-day Adventist church between February 2002 and May 2007. Participants were predominantly White (65%) and Black (27%), with a mean age of 59 y (range: 30–110 y) at enrollment. Recruitment spanned all 50 states of the United States and all Canadian provinces. At baseline, participants completed a comprehensive, validated 50-page food frequency questionnaire (FFQ) that assessed detailed dietary practices, as well as demographic information, anthropometrics, health and lifestyle behaviors (including physical activity, smoking, and alcohol use), personal and family medical history, and medication use [12]. Baseline was defined as the date the FFQ was completed during the initial enrollment period (2002–2007). Responses were carefully screened for clearly erroneous or unreliable data (e.g., uniformly high frequency responses across all food items on a page; reported diets with implausible total caloric intake or total food weight; or questionnaires with excessive missing data).

AHS-2 is unique in its broad variation in egg consumption, ranging from none to levels typical of the general United States population, and is composed of a health-conscious and aging population. For this analysis, we included only United States-based participants aged ≥65 y at enrollment. Eligibility was determined using the Medicare Master Beneficiary Summary Files. The mean follow-up period was 15.3 y.

Data from AHS-2 were match-merged with Medicare claims using a crosswalk between the AHS-2 study identifier and Medicare beneficiary identification numbers, following the approach described by Virnig et al. [13]. After merging, all data were deidentified. To ensure completeness of claims data, individuals enrolled in Medicare Advantage (managed care) plans were excluded as their claims are typically incomplete. Following guidelines from the Centers for Medicare and Medicaid Services (CMS) Chronic Conditions Data Warehouse (CCW), we excluded participants who did not have ≥10 mo of Medicare fee-for-service coverage during a calendar year.

### Exclusion criteria

The final analytical sample was restricted to Medicare beneficiaries aged ≥65 y between 2008 and 2020 who were enrolled in Medicare fee-for-service. Although individuals were eligible for enrollment beginning at age 30 y, only those who reached age 65 y during follow-up, and their person-years thereafter, were included in the present analysis. For example, individuals who were aged 63 y at enrollment were included in our analysis only after attaining age 65 y. We excluded individuals with: a diagnosis of Alzheimer’s disease at the time of AHS-2 enrollment or within 6 mo thereafter; implausible dietary data, such as energy intake of <500 or >4500 kcal/d; extreme BMI (in kg/m^2^; <16 or >60); and mismatched sex or date of birth when compared with Medicare records. After applying these criteria, the final analytical sample consisted of 39,498 participants (Supplemental Figure 1).

### Missing data

Key variables, including dietary intake and demographics, not found in Medicare Beneficiary Summary Files (e.g., marital status, education), and lifestyle factors were derived from AHS-2 baseline data. To account for missing data and ensure valid inference, we used a guided multiple imputation approach under the assumption of missing at random [14]. Five imputed datasets were created, and final estimates were obtained using Rubin’s rules by pooling parameter estimates and SEs.

### Exposure data

Dietary intake was assessed at baseline using a validated, self-administered FFQ that included >200 food items [15]. Validation against 6 24-h dietary recalls demonstrated moderate-to-high energy-adjusted correlations for egg intake: 0.64 [95% confidence interval (CI): 0.52, 0.76] for non-Black participants and 0.52 (95% CI: 0.36, 0.65) for Black participants [16]. Both the frequency and quantity of egg intake were examined. Frequency was categorized as: never/rarely, 1–3 times per month, once per week, 2–4 times per week, or ≥5 times per week based on a single FFQ item assessing visible egg consumption (e.g., boiled, scrambled, fried, deviled, omelet, or egg salad; egg-beaters were excluded). When baseline egg intake frequency was compared with a 2015 follow-up subset of >23,000 participants, ∼74% showed consistent egg consumption patterns. As previously reported [17], total egg intake was estimated using an amount variable (g/d) that captured both visible and hidden sources of eggs in the diet, including those found in mixed dishes, recipes, and baked goods. Total egg intake (g/d) was calculated and energy-adjusted using the residual method.

### Outcome ascertainment

Incident Alzheimer’s disease was identified using Medicare CCW files, which record the date beneficiaries first met claims criteria for an Alzheimer's diagnosis. Diagnoses were defined using International Classification of Diseases, Ninth Revision (ICD 9: 331.0) or Tenth Revision (ICD-10: G30.0, G30.1, G30.8, G30.9) Clinical Modification codes, following CMS CCW guidelines [18]. Alzheimer’s disease was identified if listed as a primary or coexisting condition in any available claim including inpatient, skilled nursing facility, home health agency, hospital outpatient, physician offices, or other healthcare providers. To focus on incident cases, individuals with an Alzheimer’s diagnosis prior to or within 6 mo of AHS-2 enrollment were excluded.

### Covariates

Covariates were selected a priori based on established or suspected associations with Alzheimer’s disease. They included demographics (age, sex, race, marital status, education), lifestyle variables (smoking status, alcohol use, physical activity, mean sleep duration), intake of various food groups (meat, fish, dairy, vegetables, fruits, refined grains, whole/mixed grains, legumes, nuts/seeds), and comorbidities (cardiovascular diseases, hypertension, hyperlipidemia, respiratory conditions, anemia, chronic kidney disease, hypothyroidism, and cancers, determined using Medicare Part A and Part B claims at AHS-2 enrollment). All dietary variables were energy adjusted using the residual method [16].

### Statistical analysis

Prior to analysis, the merged dataset was evaluated for outliers and scale transformations. Descriptive statistics, including means and proportions, were used to compare demographic characteristics and other covariates by egg intake category and Alzheimer’s disease status. Participants without an Alzheimer’s diagnosis were censored at the date of death (confirmed by Social Security Administration) or the last year of Medicare claim data.

Cox proportional hazards models, with attained age as the time scale and left truncation (delayed entry) applied, were used to estimate hazard ratios (HRs) and 95% CIs for the association between egg intake and incident Alzheimer’s disease using categorical (frequency) egg intake data. Participants entered the risk set at their age at AHS-2 enrollment and were followed on the attained age time scale until the earliest of: *1*) age at first qualifying Alzheimer’s disease date in the Medicare CCW; *2*) age at death; and *3*) age at end of Medicare claims availability. The Cox models were implemented using a counting-process formulation to accommodate delayed entry. The proportional hazards assumption was assessed using scaled Schoenfeld residual plots. Our Cox model estimated cause-specific hazards of Alzheimer’s disease among individuals who are alive and event-free, not population-level risk. We constructed 3 models: model 1 included demographics and lifestyle factors, including BMI; model 2, additionally adjusted for intake of major food groups; and model 3, further accounted for comorbidities. Although we considered modeling BMI as a continuous variable to reduce residual confounding, preliminary analyses indicated a nonlinear relationship with Alzheimer’s disease risk. Therefore, we retained clinically relevant categories to better capture this nonlinearity.

In addition, given the apparent nonlinearity of the association, we used restricted cubic spline models for energy-adjusted egg intake (g/d), specifying 4 knots and using 10 g/d as the reference value. Other energy-adjusted dietary variables (g/d) were modeled linearly as they did not exhibit nonlinearity.

To contextualize the associations with individual food intake [19], we conducted 2 separate substitution analyses to examine the replacement of eggs with nuts/seeds and legumes, 2 nutrient-dense, commonly consumed plant-based protein sources in our cohort. These analyses aimed to provide insight into how substituting eggs with alternative protein-rich foods might influence Alzheimer’s disease risk. The gram amounts for nuts/seeds (14 g) and legumes (60 g) were selected based on typical serving sizes, with the aim of providing approximately the same energy content as 1 large egg. In addition, to evaluate potential bias because of unmeasured systematic differences between consumers and nonconsumers, we conducted a sensitivity analysis excluding vegans. Vegans comprised a substantial portion of the zero egg consumption group, which could disproportionately influence this group, and they often differ in other lifestyle or health-related characteristics. All analyses were performed using R version 4.4.3.

### Ethical considerations

The study protocol was approved by the Institutional Review Board at Loma Linda University. Written informed consent was obtained from all participants at baseline.

## Results

Table 1 presents baseline characteristics of the 39,498 participants, stratified by frequency of egg intake. Significant differences were observed across the 5 egg intake categories for several variables, including age, sex, race, marital status, education level, BMI, physical activity, sleep duration, smoking status, and alcohol use. The mean [20] age at baseline was 64 (10.4) y and 64% (*n* = 25,171) were female. A comparison of characteristics by Alzheimer’s disease diagnosis status is presented in Supplemental Table 1. During a mean follow-up of 15.3 y, encompassing 603,754 person-y, 2858 participants were clinically diagnosed with Alzheimer’s disease. Those diagnosed with Alzheimer’s disease had a significantly higher prevalence of comorbidities. In terms of dietary intake, ∼32% of participants with Alzheimer’s disease reported no egg consumption. Compared with those without the disease, a greater proportion of Alzheimer's cases reported no meat or fish consumption, lower intake of refined grain, and higher intake of fruits, whole or mixed grains, and nuts/seeds.

**TABLE 1:** Baseline characteristics of participants in the Adventist Health Study-2 cohort by frequency of egg intake

*n*	Overall	Never	1–3/mo	1/wk	2–4/wk	5+/wk
	39,498	10,636	10,183	6938	9493	2248
Alzheimer's disease						
No	36,640 (92.8)	9724 (91.4)	9500 (93.3)	6466 (93.2)	8833 (93.0)	2117 (94.2)
Yes	2858 (7.2)	912 (8.6)	683 (6.7)	472 (6.8)	660 (7.0)	131 (5.8)
Age (y)						
65–69	6686 (16.9)	1643 (15.4)	1819 (17.9)	1201 (17.3)	1628 (17.1)	395 (17.6)
70–74	6941 (17.6)	1795 (16.9)	1836 (18.0)	1252 (18.0)	1660 (17.5)	398 (17.7)
75−79	6294 (15.9)	1669 (15.7)	1671 (16.4)	1092 (15.7)	1465 (15.4)	397 (17.7)
80–84	5624 (14.2)	1532 (14.4)	1414 (13.9)	958 (13.8)	1389 (14.6)	331 (14.7)
85–89	5037 (12.8)	1386 (13.0)	1268 (12.5)	913 (13.2)	1194 (12.6)	276 (12.3)
90–94	4282 (10.8)	1239 (11.6)	1046 (10.3)	727 (10.5)	1034 (10.9)	236 (10.5)
95+	4634 (11.7)	1372 (12.9)	1129 (11.1)	795 (11.5)	1123 (11.8)	215 (9.6)
Sex						
Male	14,327 (36.3)	3773 (35.5)	3372 (33.1)	2740 (39.5)	3503 (36.9)	939 (41.8)
Female	25,171 (63.7)	6863 (64.5)	6811 (66.9)	4198 (60.5)	5990 (63.1)	1309 (58.2)
Race and ethnicity						
Non-Hispanic White	29,352 (74.3)	7785 (73.2)	7211 (70.8)	5319 (76.7)	7228 (76.1)	1809 (80.5)
Black	7488 (19.0)	2200 (20.7)	2253 (22.1)	1092 (15.7)	1622 (17.1)	321 (14.3)
Other	2658 (6.7)	651 (6.1)	719 (7.1)	527 (7.6)	643 (6.8)	118 (5.2)
Marital status						
Married	28,910 (73.2)	7726 (72.6)	7204 (70.7)	5350 (77.1)	7011 (73.9)	1619 (72.0)
Never married	1341 (3.4)	420 (3.9)	383 (3.8)	176 (2.5)	297 (3.1)	65 (2.9)
Divorced/widowed	9247 (23.4)	2490 (23.4)	2596 (25.5)	1412 (20.4)	2185 (23.0)	564 (25.1)
Education						
High school or less	8509 (21.5)	2142 (20.1)	2328 (22.9)	1396 (20.1)	2090 (22.0)	553 (24.6)
Some college	15,589 (39.5)	4055 (38.1)	3981 (39.1)	2684 (38.7)	3898 (41.1)	971 (43.2)
College graduate	15,400 (39.0)	4439 (41.7)	3874 (38.0)	2858 (41.2)	3505 (36.9)	724 (32.2)
BMI						
Normal	15,280 (38.7)	5593 (52.6)	3845 (37.8)	2492 (35.9)	2824 (29.7)	526 (23.4)
Overweight	14,365 (36.4)	3343 (31.4)	3863 (37.9)	2698 (38.9)	3664 (38.6)	797 (35.5)
Obese	9853 (24.9)	1700 (16.0)	2475 (24.3)	1748 (25.2)	3005 (31.7)	925 (41.1)
Mean BMI (SD)	27.21 (5.46)	25.58 (4.95)	27.25 (5.36)	27.41 (5.29)	28.32 (5.61)	29.42 (5.97)
Physical activity						
None	8828 (22.4)	2117 (19.9)	2344 (23.0)	1475 (21.3)	2262 (23.8)	630 (28.0)
≤0.5 h/wk	9553 (24.2)	2196 (20.6)	2489 (24.4)	1826 (26.3)	2483 (26.2)	559 (24.9)
0.5<–2 h/wk	10,404 (26.3)	2863 (26.9)	2629 (25.8)	1866 (26.9)	2519 (26.5)	527 (23.4)
>2 h/wk	10,713 (27.1)	3460 (32.5)	2721 (26.7)	1771 (25.5)	2229 (23.5)	532 (23.7)
Sleep hours						
≤5 h	3859 (9.8)	982 (9.2)	1077 (10.6)	620 (8.9)	938 (9.9)	242 (10.8)
6 h	8611 (21.8)	2228 (20.9)	2374 (23.3)	1468 (21.2)	2041 (21.5)	500 (22.2)
7 h	14,290 (36.2)	3890 (36.6)	3582 (35.2)	2608 (37.6)	3456 (36.4)	754 (33.5)
8 h	10,486 (26.5)	2951 (27.7)	2581 (25.3)	1860 (26.8)	2496 (26.3)	598 (26.6)
≥9 h	2252 (5.7)	585 (5.5)	569 (5.6)	382 (5.5)	562 (5.9)	154 (6.9)
Smoking						
Never smoked	31,456 (79.6)	8766 (82.4)	8166 (80.2)	5597 (80.7)	7319 (77.1)	1608 (71.5)
Quit >30 y ago	3419 (8.7)	894 (8.4)	829 (8.1)	558 (8.0)	900 (9.5)	238 (10.6)
Quit 21–30 y ago	2023 (5.1)	502 (4.7)	497 (4.9)	349 (5.0)	519 (5.5)	156 (6.9)
Quit 11–20 y ago	1350 (3.4)	270 (2.5)	362 (3.6)	224 (3.2)	384 (4.0)	110 (4.9)
Quit 6–10 y ago	482 (1.2)	96 (0.9)	120 (1.2)	85 (1.2)	132 (1.4)	49 (2.2)
Quit <5 y ago	768 (1.9)	108 (1.0)	209 (2.1)	125 (1.8)	239 (2.5)	87 (3.9)
Alcohol use						
None	37,582 (95.1)	10,426 (98.0)	9721 (95.5)	6531 (94.1)	8880 (93.5)	2024 (90.0)
Current	1916 (4.9)	210 (2.0)	462 (4.5)	407 (5.9)	613 (6.5)	224 (10.0)
Cardiovascular disease						
No	34,458 (87.2)	9272 (87.2)	8913 (87.5)	6071 (87.5)	8253 (86.9)	1949 (86.7)
Yes	5040 (12.8)	1364 (12.8)	1270 (12.5)	867 (12.5)	1240 (13.1)	299 (13.3)
Hypertension						
No	32,551 (82.4)	8978 (84.4)	8404 (82.5)	5725 (82.5)	7633 (80.4)	1811 (80.6)
Yes	6947 (17.6)	1658 (15.6)	1779 (17.5)	1213 (17.5)	1860 (19.6)	437 (19.4)
Hyperlipidemia						
No	33,282 (84.3)	9032 (84.9)	8582 (84.3)	5838 (84.1)	7929 (83.5)	1901 (84.6)
Yes	6216 (15.7)	1604 (15.1)	1601 (15.7)	1100 (15.9)	1564 (16.5)	347 (15.4)
Respiratory conditions						
No	37,860 (95.9)	10,248 (96.4)	9798 (96.2)	6647 (95.8)	9037 (95.2)	2130 (94.8)
Yes	1638 (4.1)	388 (3.6)	385 (3.8)	291 (4.2)	456 (4.8)	118 (5.2)
Anemia						
No	35,746 (90.5)	9584 (90.1)	9257 (90.9)	6316 (91.0)	8547 (90.0)	2042 (90.8)
Yes	3752 (9.5)	1052 (9.9)	926 (9.1)	622 (9.0)	946 (10.0)	206 (9.2)
Chronic kidney disease						
No	38,983 (98.7)	10,525 (99.0)	10,058 (98.8)	6852 (98.8)	9340 (98.4)	2208 (98.2)
Yes	515 (1.3)	111 (1.0)	125 (1.2)	86 (1.2)	153 (1.6)	40 (1.8)
Hypothyroidism						
No	36,929 (93.5)	9959 (93.6)	9535 (93.6)	6489 (93.5)	8838 (93.1)	2108 (93.8)
Yes	2569 (6.5)	677 (6.4)	648 (6.4)	449 (6.5)	655 (6.9)	140 (6.2)
Cancers						
No	38,105 (96.5)	10,245 (96.3)	9824 (96.5)	6725 (96.9)	9137 (96.2)	2174 (96.7)
Yes	1393 (3.5)	391 (3.7)	359 (3.5)	213 (3.1)	356 (3.8)	74 (3.3)

	Mean (SD)	Mean (SD)	Mean (SD)	Mean (SD)	Mean (SD)	Mean (SD)

Meat	14.56 (25.9)	4.37 (15.1)	12.78 (22.6)	16.71 (25.5)	22.08 (29.9)	32.42 (39.6)
Fish	8.99 (15.9)	4.42 (12.9)	8.94 (15.9)	10.54 (16.3)	11.99 (16.7)	13.47 (18.8)
Dairy	149.56 (187.1)	63.85 (140.7)	152.47 (187.9)	190.84 (196.4)	199.20 (189.2)	204.80 (194.5)
Vegetables	301.52 (177.7)	342.20 (208.9)	293.17 (172.0)	289.22 (154.1)	281.84 (156.1)	267.90 (163.7)
Fruits	321.99 (221.0)	397.88 (245.6)	324.87 (222.3)	302.02 (201.0)	269.94 (182.9)	231.32 (185.7)
Refined grain	113.19 (116.3)	97.80 (115.4)	115.13 (116.7)	120.38 (115.1)	123.46 (117.7)	111.78 (109.2)
Whole/mixed grain	256.49 (188.5)	329.77 (212.3)	255.79 (188.7)	232.04 (168.4)	212.09 (153.0)	175.88 (146.7)
Nuts/seeds	23.47 (20.3)	28.32 (22.6)	22.75 (20.1)	22.29 (19.1)	20.65 (18.0)	19.28 (18.2)
Legume	77.36 (69.2)	94.01 (82.8)	76.17 (67.8)	72.80 (59.4)	67.70 (58.2)	58.90 (59.9)

*P* values were calculated using χ^2^ tests for each variable. All variables had *P* values < 0.001 except for cardiovascular disease (*P* = 0.626), hyperlipidemia (*P* = 0.108), anemia (*P* = 0.065), chronic kidney disease (*P* = 0.002), hypothyroidism (*P* = 0.492), and cancers (*P* = 0.152).

Multivariable-adjusted Cox proportional hazards models demonstrated that frequency of egg consumption was independently and inversely associated with Alzheimer’s disease risk. After adjusting for demographic characteristics, lifestyle factors, major food groups, and comorbidities, the HRs for Alzheimer’s disease, compared with never/rare egg consumption, were as follows: 0.83 (95% CI: 0.75, 0.92) for 1–3 times per month, 0.83 (0.74, 0.94) for once per week, 0.80 (0.71, 0.90) for 2–4 times per week, and 0.73 (0.60, 0.89) for 5 or more times per week. Overall, any egg intake was associated with a 17%–27% reduced risk of Alzheimer’s disease relative to no intake. This inverse association remained consistent across progressively adjusted models (Table 2).

**TABLE 2:** Multivariable-adjusted Cox proportional hazards models assessing the association between egg intake and Alzheimer’s disease risk in the Adventist Health Study-2 cohort (2008–2020; 603,754 person-y of follow-up)

	Alzheimer's cases (%)	Model 1^1^		Model 2^2^		Model 3^3^	
		HR (95% CI)	Trend *P* value	HR (95% CI)	Trend *P* value	HR (95% CI)	Trend *P* value
Egg intake			0.0002		0.0001		0.0001
Never	912 (31.9)	1.00		1.00		1.00	
1–3 times/mo	683 (23.9)	0.85 (0.77, 0.94)		0.83 (0.75, 0.93)		0.83 (0.75, 0.92)	
Once/wk	472 (16.5)	0.87 (0.77, 0.97)		0.85 (0.75, 0.95)		0.83 (0.74, 0.94)	
2–4 times/wk	660 (23.1)	0.83 (0.75, 0.92)		0.81 (0.72, 0.90)		0.80 (0.71, 0.90)	
>5 times/wk	131 (4.6)	0.76 (0.63, 0.92)		0.73 (0.60, 0.89)		0.73 (0.60, 0.89)	

Abbreviations: CI, confidence interval; HR, hazard ratio.

1 Model 1: adjusted for: sex (male, female), race (non-Hispanic White, Black, other), marital status (married, never married, divorced/widowed), educational level (high school or less, some college, college graduate or more), BMI (<25, 25–29.9, ≥30 kg/m^2^), physical activity (none, ≤0.5, 0.5–<2, >2 h/wk), sleep duration (≤5, 6, 7, 8, ≥9 h) smoking status (never, quit ≥30, 21–29, 11–20, 6–10, <1 to 5 y ago), alcohol use (none, current), and total energy intake (per 100 kcal/d).

2 Model 2: adjusted for all variables in model 1 plus energy-adjusted intake (g/d) of major food groups: meat, fish, dairy, vegetables, fruits, refined grains, whole/mixed grains, nuts/seeds, and legumes.

3 Model 3: adjusted for all variables in model 2 plus comorbid conditions: cardiovascular diseases, hypertension, hyperlipidemia, respiratory conditions, anemia, chronic kidney disease, hypothyroidism, and cancers (each categorized as yes/no).

An additional analysis isolating individuals who consumed 1 or more eggs per day is presented in Supplemental Table 2. The HR for consuming eggs once per day or more, compared with never/rare consumption, was 0.74 (0.55, 0.98). However, the small number of participants in this group limited statistical power. Therefore, the primary analyses retained the original intake categories to preserve model stability. No significant interactions by sex or race/ethnicity were observed. Because attained age was used as the time variable in the Cox models, age-related risk was inherently accounted for. Results from the sensitivity analysis excluding vegans remained very consistent with the primary findings (Table 3). The HR for consuming eggs 5 or more times/wk, compared with never/rare consumption, was 0.73 (0.60, 0.89).

**TABLE 3:** Multivariable-adjusted Cox proportional hazards models assessing the association between egg intake and Alzheimer’s disease risk excluding vegans (*n* = 3263) in the Adventist Health Study-2 cohort (2008–2020): total *N* = 36,235

	Alzheimer's cases (%)	Model 1^1^				Model 2^2^				Model 3^3^			
		HR	95% CI		Trend	HR	95% CI		Trend	HR	95% CI		Trend
			Lower	Upper	*P* value		Lower	Upper	*P* value		Lower	Upper	*P* value
Egg intake					0.0009				0.0004				0.0004
Never	638	1.00				1.00				1.00			
1–3 times/mo	683	0.84	0.76	0.94		0.83	0.75	0.93		0.83	0.75	0.93	
Once/wk	472	0.86	0.76	0.97		0.85	0.75	0.96		0.84	0.74	0.95	
2–4 times/wk	660	0.82	0.74	0.92		0.81	0.72	0.91		0.80	0.71	0.91	
5+ times/wk	131	0.76	0.62	0.92		0.73	0.60	0.89		0.73	0.60	0.89	

Abbreviations: CI, confidence interval; HR, hazard ratio.

1 Model 1: adjusted for: sex (male, female), race (non-Hispanic White, Black, other), marital status (married, never married, divorced/widowed), educational level (high school or less, some college, college graduate or more), BMI (<25, 25–29.9, ≥30 kg/m^2^), physical activity (none, ≤0.5, 0.5–<2, >2 h/wk), sleep duration (≤5, 6, 7, 8, ≥9 h) smoking status (never, quit ≥30, 21–29, 11–20, 6–10, <1 to 5 y ago), alcohol use (none, current), and total energy intake (per 100 kcal/d)

2 Model 2: adjusted for all variables in model 1 plus energy-adjusted intake (g/d) of major food groups: meat, fish, dairy, vegetables, fruits, refined grains, whole/mixed grains, nuts/seeds, and legumes.

3 Model 3: adjusted for all variables in model 2 plus comorbid conditions: cardiovascular diseases, hypertension, hyperlipidemia, respiratory conditions, anemia, chronic kidney disease, hypothyroidism, and cancers (each categorized as yes/no).

**FIGURE 1:**
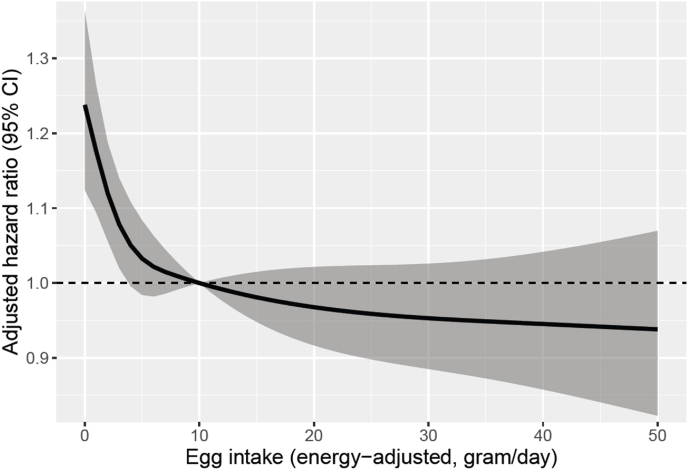
Restricted cubic spline model depicting the association between energy-adjusted egg intake (g/d) and risk of Alzheimer’s disease. Adjusted HRs are estimated with 10 g/d as the reference value; shaded areas represent 95% CI. CI, confidence interval; HR, hazard ratio.

Figure 1 displays a restricted cubic spline model of energy-adjusted egg intake (g/d), illustrating a nonlinear inverse relationship between egg consumption and Alzheimer’s disease risk. Compared with an intake of 10 g/d (∼1 large egg per week), participants with zero egg intake had an adjusted HR of 1.22 (95% CI: 1.11, 1.34), indicating a significantly elevated risk. Although the risk declined progressively with increasing egg intake beyond 10 g/d, these differences were not statistically significant. Results from the substitution analyses were highly consistent with one another and aligned closely with the main findings (Table 4). Substituting eggs for nuts/seeds or legumes in the diet was associated with a similarly reduced risk of Alzheimer’s disease.

**TABLE 4:** Association between substituting eggs for nuts/seeds and legumes and the risk of Alzheimer's disease in the AHS-2 cohort

	Model 1^1^			Model 2^2^		
	HR	95% CI		HR	95% CI	
Eggs 1–3 times/mo for 2 svg/mo of nuts/seeds	0.83	0.75	0.93	0.83	0.75	0.92
Eggs once/wk for 1 svg/wk of nuts/seeds	0.85	0.75	0.95	0.83	0.74	0.94
Eggs 2–4 times/wk for 3 svg/wk of nuts/seeds	0.80	0.72	0.90	0.80	0.71	0.89
Eggs 5+ times/wk 1 svg/d of nuts/seeds	0.73	0.59	0.89	0.72	0.59	0.88
Eggs 1–3 times/mo for 2 svg/mo of legumes	0.84	0.75	0.93	0.83	0.75	0.92
Eggs once/wk for 1 svg/wk of legumes	0.85	0.75	0.95	0.84	0.74	0.94
Eggs 2–4 times/wk for 3 svg/wk of legumes	0.82	0.73	0.92	0.81	0.73	0.91
Eggs 5+ times/wk for 1 svg/d of legumes	0.75	0.62	0.92	0.75	0.62	0.92

Abbreviations: AHS-2, Adventist Health Study-2; CI, confidence interval; HR, hazard ratio; Svg, servings.

One serving is defined as 14 g of nuts and seeds and 60 g of legumes.

1 Model 1: adjusted for: sex (male, female), race (non-Hispanic White, Black, other), marital status (married, never married, divorced/widowed), educational level (high school or less, some college, college graduate or more), BMI (<25, 25–29.9, ≥30 kg/m^2^), physical activity (none, ≤0.5, 0.5–<2, >2 h/wk), sleep duration (≤5, 6, 7, 8, ≥9 h) smoking status (never, quit ≥30, 21–29, 11–20, 6–10, <1–5 y ago), alcohol use (none, current), and total energy intake (per 100 kcal/d), and energy-adjusted intake (g/d) of major food groups: meat, fish, dairy, vegetables, fruits, refined grains, whole/mixed grains, and legumes (for nuts/seeds substitution only) or nuts/seeds (for legumes substitution only).

2 Model 2: adjusted for all variables in model 1 plus comorbid conditions: cardiovascular diseases, hypertension, hyperlipidemia, respiratory conditions, anemia, chronic kidney disease, hypothyroidism, and cancers (each categorized as yes/no).

## Discussion

We found that moderate egg intake was independently associated with a lower incidence of Alzheimer's disease, even after adjusting for other dietary factors, demographic variables, lifestyle behaviors, and comorbidities. This study builds on our previous findings [10] by isolating the independent association between egg consumption and Alzheimer’s disease, controlling for intake of other food groups. Notably, egg consumption in our cohort was lower than that of the general United States population, which may have led to an underestimation of the observed protective association. This, in turn, further supports the plausibility of a beneficial effect.

Eggs are a rich source of nutrients relevant to brain health. They provide choline, a precursor to acetylcholine and phosphatidylcholine, both of which are critical for memory and synaptic function [11]. Eggs also contain lutein and zeaxanthin-carotenoids that accumulate in brain tissue and are associated with improved cognitive performance and reduced oxidative stress [9,11]. Other key nutrients in eggs include high-quality protein rich in tryptophan (a serotonin precursor involved in mood regulation, cognition, and melatonin synthesis) and DHA [21], an omega-3 (n–3) fatty acid important for synaptic plasticity, neurogenesis, and neuronal membrane integrity [9,11,21]. Emerging evidence also highlights the role of egg-derived tryptophan peptides in enhancing attention, reducing stress reactivity, and improving executive function in older adults [11]. These nutrients may act synergistically to support cognitive resilience and mitigate neurodegenerative processes. Notably, deficiencies in choline and DHA have been documented in the brains of individuals with Alzheimer’s disease [21,22].

Egg yolks are particularly rich in phospholipids, which constitute nearly 30% of total egg lipids and are essential for neurotransmitter receptor function [11]. Eggs also contain choline and DHA—both critical for acetylcholine synthesis, neurogenesis, and synaptic maintenance [11]. Although both choline and DHA can be effectively delivered to the brain through dietary intake, their transport across the blood–brain barrier and overall bioavailability may differ [23]. Although egg consumption has historically been limited because of concerns about cholesterol, recent meta-analyses and systematic reviews indicate that moderate egg consumption does not adversely impact cardiovascular or metabolic health in most individuals, especially when consumed as part of a balanced diet [24].

In addition, there is growing evidence that vitamin B12 is a key nutrient for brain health. Eggs, particularly yolks, are a good dietary source, with 1 egg providing ∼25% of the recommended dietary allowance of vitamin B12, which is often limited in vegetarian diets. Vitamin B12 plays a multifaceted role in brain function and the pathogenesis of Alzheimer’s disease through its involvement in one-carbon metabolism, amyloid and tau regulation, oxidative balance, inflammation, lipid metabolism, and mitochondrial function [25]. Deficiency can elevate homocysteine, a recognized Alzheimer’s disease risk factor, and promote neuroinflammation through altered methylation and cytokine activity [25]. In the AHS-2 cohort, we found that, overall, all dietary groups had adequate vitamin B12 levels. However, a small proportion of nonsupplementing vegetarians may have had insufficient levels [26].

Despite the biological plausibility of a protective role, few studies have directly examined the relationship between egg consumption and incident Alzheimer’s disease. A population-based case-control study in Chinese adults reported significantly lower odds of dementia among daily egg consumers compared with less frequent consumers [9]. In a United States cohort of older females, higher egg intake was associated with less decline in semantic memory and executive function over a 4-y period [27]. Similarly, our previous study found that moderate egg consumption (∼1 egg per week) was linked to a slower rate of memory decline, although a dose–response relationship was not apparent [10]. These findings suggest that regular but not excessive egg intake may support cognitive maintenance.

In a Finnish prospective study of 2497 middle-aged males followed over 2 decades, moderate egg consumption was associated with better neuropsychological test performance and did not increase Alzheimer’s disease risk [28]. Similarly, in the European Prospective Investigation into Cancer Nutrition (EPIC-Spain) dementia cohort, which included 774 dementia cases, higher whole egg intake was associated with lower dementia risk [29]. Data from the Rush Memory and Aging project with 280 Alzheimer’s cases showed that consuming ≥1 egg per week was associated with a 46% reduced risk of Alzheimer’s disease [22]. The Framingham Heart Study further reported that the highest levels of phosphatidylcholine-DHA were significantly associated with a reduced risk of all-cause dementia [30].

This study demonstrates a significant, independent inverse association between egg consumption and Alzheimer’s disease risk within a health-conscious cohort with low prevalence of traditional risk factors (e.g., 80% never-smokers). These findings strengthen evidence supporting the potential neuroprotective benefit of egg consumption within the context of a balanced dietary pattern. This contrasts with results from the EPIC-Spain study, where a protective association was observed only among individuals with low adherence to the Mediterranean diet. Given that Alzheimer’s disease pathology often begins ≤20 y before clinical symptoms appear [1], identifying modifiable lifestyle factors, such as diet, is critical. With the rapid aging of the United States population and projected increases in healthcare costs, understanding the potential role of egg consumption in reducing Alzheimer’s risk carries important implications, especially for Medicare, the largest source of healthcare spending in the United States.

### Strengths and limitations

This study has several notable strengths. Its prospective, longitudinal design, long follow-up period, large number of clinically ascertained Alzheimer’s disease cases, and wide range of egg intake all contribute to the robustness of our findings. Over 15 y of follow-up and the use of a validated FFQ allowed for a high-quality assessment of habitual dietary intake. In addition, Alzheimer’s disease and comorbidities were identified through Medicare data enhancing disease ascertainment. A validation study reported 87%–90% accuracy for ICD-10-based Alzheimer’s disease algorithms in Medicare data [31]. The large number of confirmed Alzheimer’s cases within the AHS-2 cohort provided strong statistical power, enabling detection of associations with individual dietary components. Furthermore, the cohort’s low prevalence of smoking and alcohol use reduces potential confounding from these established risk factors: ∼80% were never-smokers, and few reported alcohol use. A notable methodological strength is the use of restricted cubic spline modeling to quantify nonlinear risk across the full range of egg intake. The approach yielded a novel public health finding that zero egg intake was curvilinearly associated with significantly elevated Alzheimer’s disease risk. Lastly, to address missing data, we used a guided multiple imputation approach.

Nonetheless, several limitations should be acknowledged. First, as with all Medicare-based studies, we could not determine whether participants aged ≥65 y were actively using Medicare as their primary insurance. However, given that 96% of United States adults aged ≥65 y are enrolled in Medicare, the risk of substantial bias is low. Second, dietary intake was assessed only at baseline. It is possible that dietary changes occurred during the preclinical phase of Alzheimer’s disease, or following other diagnoses, potentially leading to reverse causation. However, prior research within this cohort has demonstrated notable stability in dietary patterns over time, particularly among older adults [32]. In our own follow-up data, egg intake showed a high degree of consistency, supporting the validity of using baseline dietary data to infer long-term associations with Alzheimer’s disease risk. Therefore, reverse causation is unlikely to have influenced the observed associations. In addition, Medicare claims data may underestimate the identification of individuals with Alzheimer’ disease, particularly those with milder or less clinically apparent symptoms. Although the highest category of egg intake (≥5 times per week) showed the greatest risk reduction, the small number of participants consuming ≥1 egg per day limited our ability to assess higher intake levels. Another limitation is the semicompeting risks structure inherent in studies of Alzheimer’s disease in older adults. Because death is a terminal event that may be related to the nonterminal event of Alzheimer’s diagnosis, treating death as noninformative censoring can constrain the interpretation of the HRs, particularly when considering absolute risk or incidence. Our Cox models estimated associations with the instantaneous rate of Alzheimer’s diagnosis among individuals who are alive and event-free, and these estimates should not be interpreted as reflecting differences in absolute risk in the presence of potential differential mortality [33]. Lastly, despite extensive adjustment for demographic and lifestyle factors, residual confounding cannot be entirely ruled out in observational studies of this nature.

This study examined whether habitual egg consumption is associated with incident Alzheimer’s disease in a well-characterized United States cohort. Leveraging a large sample size, prospective design, and a clinically confirmed outcome, our findings provide insight into the potential neuroprotective role of whole-food nutrient sources such as eggs. Further research is warranted to explore this relationship in more diverse populations, evaluate whether long-term egg consumption earlier in life influences later risk of Alzheimer’s disease, and to investigate the role of specific egg-derived nutrients in relation to that risk.

## Author contributions

The authors’ responsibilities were as follows – JO, JS: designed and conducted the research and primary responsible for the final content; JS, GEF: provided essential materials; KO, JO: performed the data analyses; JO: drafted the initial version of the manuscript; GC: contributed to specific sections; RS: reviewed and edited the manuscript; and all authors: reviewed, revised, and approved the final version of the manuscript.

## Data availability

The data described in the manuscript are available on request, pending application and approval.

## Declaration of Generative AI and AI-assisted technologies in the writing process

During the preparation of this work, the authors used ChatGPT in order to assist with word choice and phrasing. After using this tool/service, the authors reviewed and edited the content as needed and take full responsibility for the content of the publication.

## Funding

Initial support for the cohort was provided by the National Cancer Institute (grant 1U01CA152939). The analyses in this study were supported by an investigator-initiated grant from the American Egg Board. The funding sources had no role in the study design, execution, data analysis, interpretation, manuscript preparation, or publication.

## Conflict of interest

The authors report no conflicts of interest.
