# Supplementary material for: Egg Intake and the Incidence of Alzheimer’s Disease in the Adventist Health Study-2 Cohort Linked with Medicare Data
Source: J Nutr. 2026 Apr 17;156(6):101541. doi: 10.1016/j.tjnut.2026.101541 (PMC13279293; doi:10.1016/j.tjnut.2026.101541)
Supplement: Multimedia component 1 [file mmc1.docx]

**Supplemental Figure 1**. Participant Flowchart

Final analytic sample

N=39,498

Participants with matched Medicare data

(n=41,041)

Total AHS-2 participants

(n=95,863)

Excluded participants with missing or partial social security numbers

(n=24,895)

Available for Medicare matching

(n=70,968)

Excluded participants with no matching Medicare record (e.g., Canadians, duplicate records, or discrepancies in sex or date of birth) and those with implausible dietary data

(n=29,927)

Further exclusions:

- Individuals providing no person-years under age 65 (n=1,332)
- BMI <16 or >60 (n=83)
- Prevalent Alzheimer’s disease at baseline (n=109)
- Unverified dates of death (n=19)

**Supplemental Table 1.** Baseline characteristics of participants in the Adventist Health Study-2 cohort

|  |  | **Overall** | **Non-case** | **Case** |
| --- | --- | --- | --- | --- |
| **n** |  | 39498 | 36640 | 2858 |
| **Egg Intake** | Never | 10636 (26.9) | 9724 (26.5) | 912 (31.9) |
|  | 1-3/mo | 10183 (25.8) | 9500 (25.9) | 683 (23.9) |
|  | 1/wk | 6938 (17.6) | 6466 (17.6) | 472 (16.5) |
|  | 2-4/wk | 9493 (24.0) | 8833 (24.1) | 660 (23.1) |
|  | 5+/wk | 2248 ( 5.7) | 2117 ( 5.8) | 131 ( 4.6) |
| **Age*** | 65-69 | 6686 (16.9) | 6664 (18.2) | 22 ( 0.8) |
|  | 70-74 | 6941 (17.6) | 6869 (18.7) | 72 ( 2.5) |
|  | 75-79 | 6294 (15.9) | 6108 (16.7) | 186 ( 6.5) |
|  | 80-84 | 5624 (14.2) | 5264 (14.4) | 360 (12.6) |
|  | 85-89 | 5037 (12.8) | 4467 (12.2) | 570 (19.9) |
|  | 90-94 | 4282 (10.8) | 3549 ( 9.7) | 733 (25.6) |
|  | 95+ | 4634 (11.7) | 3719 (10.2) | 915 (32.0) |
| **Sex** | Male | 14327 (36.3) | 13385 (36.5) | 942 (33.0) |
|  | Female | 25171 (63.7) | 23255 (63.5) | 1916 (67.0) |
| **Race** | NH White | 29352 (74.3) | 27022 (73.8) | 2330 (81.5) |
|  | Black | 7488 (19.0) | 7053 (19.2) | 435 (15.2) |
|  | Other | 2658 ( 6.7) | 2565 ( 7.0) | 93 ( 3.3) |
| **Marital Status** | Married | 28910 (73.2) | 27052 (73.8) | 1858 (65.0) |
|  | Never married | 1341 ( 3.4) | 1255 ( 3.4) | 86 ( 3.0) |
|  | Divorced/Widowed | 9247 (23.4) | 8333 (22.7) | 914 (32.0) |
| **Education** | High school or less | 8509 (21.5) | 7694 (21.0) | 815 (28.5) |
|  | Some college | 15589 (39.5) | 14500 (39.6) | 1089 (38.1) |
|  | College graduate | 15400 (39.0) | 14446 (39.4) | 954 (33.4) |
| **BMI** | Normal | 15280 (38.7) | 14000 (38.2) | 1280 (44.8) |
|  | Overweight | 14365 (36.4) | 13335 (36.4) | 1030 (36.0) |
|  | Obese | 9853 (24.9) | 9305 (25.4) | 548 (19.2) |
| **Mean BMI** |  | 27.21 (5.46) | 27.27 (5.48) | 26.41 (5.18) |
| **Physical Activity** | None | 8828 (22.4) | 7974 (21.8) | 854 (29.9) |
|  | ≤0.5 hrs/wk | 9553 (24.2) | 9015 (24.6) | 538 (18.8) |
|  | 0.5<-2 hrs/wk | 10404 (26.3) | 9717 (26.5) | 687 (24.0) |
|  | >2 hrs/wk | 10713 (27.1) | 9934 (27.1) | 779 (27.3) |
| **Sleep Duration** | <= 5 hrs | 3859 ( 9.8) | 3611 ( 9.9) | 248 ( 8.7) |
|  | 6 hrs | 8611 (21.8) | 8040 (21.9) | 571 (20.0) |
|  | 7 hrs | 14290 (36.2) | 13361 (36.5) | 929 (32.5) |
|  | 8 hrs | 10486 (26.5) | 9604 (26.2) | 882 (30.9) |
|  | >= 9 hrs | 2252 ( 5.7) | 2024 ( 5.5) | 228 ( 8.0) |
| **Smoking** | Never | 31456 (79.6) | 29141 (79.5) | 2315 (81.0) |
|  | Quit >30 yrs | 3419 ( 8.7) | 3088 ( 8.4) | 331 (11.6) |
|  | Quit 21-30 yrs | 2023 ( 5.1) | 1925 ( 5.3) | 98 ( 3.4) |
|  | Quit 11-20 yrs | 1350 ( 3.4) | 1287 ( 3.5) | 63 ( 2.2) |
|  | Quit 6-10 yrs | 482 ( 1.2) | 462 ( 1.3) | 20 ( 0.7) |
|  | Quit <5 yrs | 768 ( 1.9) | 737 ( 2.0) | 31 ( 1.1) |
| **Alcohol Use** | None | 37582 (95.1) | 34804 (95.0) | 2778 (97.2) |
|  | Current | 1916 ( 4.9) | 1836 ( 5.0) | 80 ( 2.8) |
| **Depression** | No | 37936 (96.0) | 35403 (96.6) | 2533 (88.6) |
|  | Yes | 1562 ( 4.0) | 1237 ( 3.4) | 325 (11.4) |
| **Func. Disability** | No | 29755 (75.3) | 28624 (78.1) | 1131 (39.6) |
|  | Yes | 9743 (24.7) | 8016 (21.9) | 1727 (60.4) |
| **Diabetes** | No | 37131 (94.0) | 34655 (94.6) | 2476 (86.6) |
|  | Yes | 2367 ( 6.0) | 1985 ( 5.4) | 382 (13.4) |
| **CVD** | No | 34458 (87.2) | 32488 (88.7) | 1970 (68.9) |
|  | Yes | 5040 (12.8) | 4152 (11.3) | 888 (31.1) |
| **Hypertension** | No | 32551 (82.4) | 30856 (84.2) | 1695 (59.3) |
|  | Yes | 6947 (17.6) | 5784 (15.8) | 1163 (40.7) |
| **Hyperlipidemia** | No | 33282 (84.3) | 31438 (85.8) | 1844 (64.5) |
|  | Yes | 6216 (15.7) | 5202 (14.2) | 1014 (35.5) |
| **Respiratory Conditions** | No | 37860 (95.9) | 35266 (96.2) | 2594 (90.8) |
|  | Yes | 1638 ( 4.1) | 1374 ( 3.8) | 264 ( 9.2) |
| **Anemia** | No | 35746 (90.5) | 33596 (91.7) | 2150 (75.2) |
|  | Yes | 3752 ( 9.5) | 3044 ( 8.3) | 708 (24.8) |
| **Chronic Kidney Disease** | No | 38983 (98.7) | 36200 (98.8) | 2783 (97.4) |
|  | Yes | 515 ( 1.3) | 440 ( 1.2) | 75 ( 2.6) |
| **Hypothyroidism** | No | 36929 (93.5) | 34557 (94.3) | 2372 (83.0) |
|  | Yes | 2569 ( 6.5) | 2083 ( 5.7) | 486 (17.0) |
| **Cancers** | No | 38105 (96.5) | 35463 (96.8) | 2642 (92.4) |
|  | Yes | 1393 ( 3.5) | 1177 ( 3.2) | 216 ( 7.6) |
| **Meat Intake** | None | 19831 (50.2) | 18255 (49.8) | 1576 (55.1) |
|  | <11 g/d | 6626 (16.8) | 6119 (16.7) | 507 (17.7) |
|  | 11-<32 g/d | 6482 (16.4) | 6061 (16.5) | 421 (14.7) |
|  | 32+ g/d | 6559 (16.6) | 6205 (16.9) | 354 (12.4) |
| **Fish Intake** | None | 19356 (49.0) | 17826 (48.7) | 1530 (53.5) |
|  | <9 g/d | 7122 (18.0) | 6566 (17.9) | 556 (19.5) |
|  | 9-<18 g/d | 6766 (17.1) | 6329 (17.3) | 437 (15.3) |
|  | 18+ g/d | 6254 (15.8) | 5919 (16.2) | 335 (11.7) |
| **Dairy Intake** | None | 4501 (11.4) | 4141 (11.3) | 360 (12.6) |
|  | <50 g/d | 11404 (28.9) | 10590 (28.9) | 814 (28.5) |
|  | 50-<180 g/d | 11690 (29.6) | 10900 (29.7) | 790 (27.6) |
|  | 180+ g/d | 11903 (30.1) | 11009 (30.0) | 894 (31.3) |
| **Vegetable Intake** | <185 g/d | 10054 (25.5) | 9352 (25.5) | 702 (24.6) |
|  | 185-<270 g/d | 9753 (24.7) | 9028 (24.6) | 725 (25.4) |
|  | 270-<380 g/d | 9939 (25.2) | 9204 (25.1) | 735 (25.7) |
|  | 380+ g/d | 9752 (24.7) | 9056 (24.7) | 696 (24.4) |
| **Fruit Intake** | <170 g/d | 9909 (25.1) | 9333 (25.5) | 576 (20.2) |
|  | 170-<280 g/d | 9811 (24.8) | 9152 (25.0) | 659 (23.1) |
|  | 280-<420 g/d | 9931 (25.1) | 9111 (24.9) | 820 (28.7) |
|  | 420+ g/d | 9847 (24.9) | 9044 (24.7) | 803 (28.1) |
| **Refined Grain Intake** | <40 g/d | 10362 (26.2) | 9405 (25.7) | 957 (33.5) |
|  | 40-<83 g/d | 9981 (25.3) | 9252 (25.3) | 729 (25.5) |
|  | 83-<150 g/d | 9771 (24.7) | 9152 (25.0) | 619 (21.7) |
|  | 150+ g/d | 9384 (23.8) | 8831 (24.1) | 553 (19.3) |
| **Whole/Mixed Grain Intake** | <120 g/d | 10209 (25.8) | 9605 (26.2) | 604 (21.1) |
|  | 120-<210 g/d | 9621 (24.4) | 8943 (24.4) | 678 (23.7) |
|  | 210-<350 g/d | 9681 (24.5) | 8920 (24.3) | 761 (26.6) |
|  | 350+ g/d | 9987 (25.3) | 9172 (25.0) | 815 (28.5) |
| **Nuts/Seeds Intake** | <9 g/d | 9917 (25.1) | 9290 (25.4) | 627 (21.9) |
|  | 9-<19 g/d | 10329 (26.2) | 9636 (26.3) | 693 (24.2) |
|  | 19-<33 g/d | 9789 (24.8) | 9068 (24.7) | 721 (25.2) |
|  | 33+ g/d | 9463 (24.0) | 8646 (23.6) | 817 (28.6) |
| **Legume Intake** | <33 g/d | 9699 (24.6) | 8990 (24.5) | 709 (24.8) |
|  | 33-<60 g/d | 9671 (24.5) | 8903 (24.3) | 768 (26.9) |
|  | 60-<100 g/d | 10386 (26.3) | 9651 (26.3) | 735 (25.7) |
|  | 100+ g/d | 9742 (24.7) | 9096 (24.8) | 646 (22.6) |

p-values were calculated using Chi-square tests for each variable. All variables had p-values <0.001 except for dairy (p=0.349), vegetables (p=0.906), and legumes (p=0.002)

*Age at the end of the study period (2020), when the most recent Medicare data were available for data analysis

BMI: body mass index

Func. Disability: functional disability

CVD: cardiovascular disease

**Supplemental Table 2.** Multivariable-adjusted Cox proportional hazard models assessing the association between egg intake and Alzheimer’s disease risk, with a breakdown of the highest intake category in the Adventist Health Study-2 cohort (2008-2020)

|  |  | **Model 1^1^** | | | | **Model 2^2^** | | | | **Model 3^3^** | | | |
| --- | --- | --- | --- | --- | --- | --- | --- | --- | --- | --- | --- | --- | --- |
|  |  |  | **95% CI** | | **Trend** |  | **95% CI** | | **Trend** |  | **95% CI** | | **Trend** |
|  | **Alzheimer's Cases (%)** | **HR** | **Lower** | **Upper** | **p-value** | **HR** | **Lower** | **Upper** | **p-value** | **HR** | **Lower** | **Upper** | **p-value** |
| **Egg Intake** |  |  |  |  | 0.0003 |  |  |  | 0.0001 |  |  |  | <.0001 |
| Never | 912 (31.9) | 1.00 |  |  |  | 1.00 |  |  |  | 1.00 |  |  |  |
| 1-3 times/month | 683 (23.9) | 0.85 | 0.77 | 0.94 |  | 0.83 | 0.75 | 0.93 |  | 0.83 | 0.75 | 0.92 |  |
| once/week | 472 (16.5) | 0.87 | 0.77 | 0.97 |  | 0.85 | 0.75 | 0.95 |  | 0.83 | 0.74 | 0.94 |  |
| 2-4 times/week | 660 (23.1) | 0.83 | 0.75 | 0.92 |  | 0.81 | 0.72 | 0.90 |  | 0.80 | 0.71 | 0.90 |  |
| 5-6 times/week | 79 (0.03) | 0.76 | 0.60 | 0.97 |  | 0.73 | 0.57 | 0.93 |  | 0.72 | 0.56 | 0.92 |  |
| once/day or more | 52 (0.02) | 0.77 | 0.58 | 1.02 |  | 0.73 | 0.54 | 0.97 |  | 0.74 | 0.55 | 0.98 |  |

**^1^ Model 1**: Adjusted for: sex (male, female), race (Non-Hispanic White, Black, other), marital status (married, never married, divorced/widowed), educational level (high school or less, some college, college graduate or more), body mass index (<25, 25-29.9, >=30 kg/m^2^), physical activity (none, <=0.5 hours/week, 0.5-<2 hours/week, >2 hours/week), sleep duration (<=5,, 6, 7, 8, >=9 hours) smoking status (never, quit >=30 years ago, 21-29 years ago, 11-20 years ago, 6-10 years ago, <1 to 5 year ago), alcohol use (none, current), and total energy intake (per 100 kcal/day)

**^2^ Model 2**: Adjusted for all variables in Model 1 plus energy-adjusted intake (g/day) of major food groups: meat, fish, dairy, vegetables, fruits, refined grains, whole/mixed grains, nuts/seeds, and legumes

**^3^ Model 3**: Adjusted for all variables in Model 2 plus comorbid conditions: cardiovascular diseases, hypertension, hyperlipidemia, respiratory conditions, anemia, chronic kidney disease, hypothyroidism, and cancers (each categorized as yes/no)

HR: hazard ratio

CI: confidence interval
